# Diagnostic performance of vascular endothelial growth factor D in severity stratification of pediatric COVID-19 and multisystem inflammatory syndrome in children

**DOI:** 10.3389/fimmu.2026.1799910

**Published:** 2026-07-08

**Authors:** Kateryna Kozak, Halyna Pavlyshyn

**Affiliations:** Department of Pediatrics No. 2, I. Horbachevsky Ternopil National Medical University, Ternopil, Ukraine

**Keywords:** VEGF-D, endothelial dysfunction, inflammation, COVID-19, MIS-C, children

## Abstract

**Introduction:**

In recent years, vascular endothelial growth factor D (VEGF-D) has been recognized as a promising biomarker for disease severity stratification in coronavirus disease, as well as an indicator of endothelial dysfunction, hypoxia, and pathological angiogenesis − all of which are hallmarks of severe COVID-19. However, there is limited evidence on the specific role of VEGF-D in children with COVID-19, and its relevance in multisystem inflammatory syndrome in children (MIS-C) has not been thoroughly explored.

**Methods:**

This study included 200 children with confirmed COVID-19 (through PCR or rapid antigen test), 40 children with MIS-C, and 24 healthy children without SARS-CoV-2 infection. Serum VEGF-D concentrations, acute-phase markers (C-reactive protein, procalcitonin, ferritin), and interleukin levels (IL-1β, IL-6, TNF-α) were measured using enzyme-linked immunosorbent assay (ELISA) with commercial kits.

**Results:**

The results showed that VEGF-D levels were significantly higher in children with COVID-19 (325.61 (189.93; 535.85) pg/ml) and MIS-C (920.92 (473.45; 1157.70) pg/ml) compared with the control group (195.88 (115.58; 256.70) pg/ml), with the highest levels observed in patients with MIS-C and severe COVID-19. The increase in VEGF-D was more prominent in boys infected by SARS-CoV-2, but there were no significant differences based on age within the groups. Multivariable logistic regression analysis showed that VEGF-D was independently associated with severe COVID-19 and MIS-C in children. Receiver operating characteristic (ROC) analysis established clinically relevant cut-off values for VEGF-D: 387.87 pg/mL for identifying severe COVID-19 and 461.96 pg/mL for differentiating MIS-C from acute COVID-19. In children with COVID-19, exceeding the VEGF-D cut-off was associated with more severe hypoxia and higher levels of C-reactive protein and ferritin, while in MIS-C it was associated with elevated CRP and IL-6. Principal component analysis showed that VEGF-D, along with acute-phase markers and pro-inflammatory cytokines, contributes to an integrated inflammatory-endothelial profile in both COVID-19 and MIS-C.

**Conclusion:**

VEGF-D is an independent marker associated with disease severity in pediatric COVID-19 and MIS-C and may be useful for severity stratification, differentiating clinical phenotypes, and informing patient management.

## 1 Introduction

Vascular endothelial growth factor D (VEGF-D) belongs to the VEGF family, a group of glycoproteins with central roles in vascular biology, including VEGF-A, VEGF-B, VEGF-C, VEGF-D, and placental growth factor (PlGF) (1–4). VEGF is produced by various cell types, such as macrophages, keratinocytes, mesangial cells, and endothelial cells, and was initially described as a vascular permeability factor; but subsequent studies established its mitogenic and angiogenic properties (1, 5–7). While VEGF family members have partially overlapping effects, they differ in receptor affinity, downstream biological functions, and potential clinical relevance (3, 6). VEGF-A primarily mediates classical angiogenic and vascular permeability responses, as well as endothelial cell proliferation, through VEGFR-1 and VEGFR-2 (3, 8). VEGF-B mainly binds to VEGFR-1 and neuropilin-1 (NRP-1) and is involved in angiocrine signaling, which facilitates communication between endothelial cells and neighboring tissues (8, 9). Recent data have shown that VEGF-B may also have regulatory or anti-angiogenic effects by inhibiting the FGF2/FGFR1 pathway, highlighting the importance of not considering VEGF family members as interchangeable markers (10). VEGF-C and VEGF-D are more closely linked to VEGFR-3-mediated lymphangiogenesis, lymphatic endothelial remodeling, tissue-fluid drainage, and regulation of immune-cell trafficking (3, 11). Experimental data also support the immunoregulatory relevance of the VEGF-C/VEGFR-3 axis in lung injury, as VEGF-C/VEGFR-3 signaling in macrophages was shown to limit inflammation and promote efferocytosis during the resolution of acute lung injury (12, 13). After proteolytic processing, VEGF-D can also interact with VEGFR-2, connecting lymphangiogenesis with angiogenesis, endothelial remodeling, and changes in microvascular permeability (3). Together, these receptor- and pathway-specific features provide a biological rationale for evaluating VEGF-D separately from total VEGF or other VEGF-family members.

VEGF-D plays a crucial role in lymphangiogenesis and vascular remodeling by promoting the growth and remodeling of both lymphatic and blood vessels (2, 3). Under physiological conditions, it supports the structure and function of the lymphatic system by facilitating immune-cell trafficking, promoting angiogenesis and lymphangiogenesis, and regulating microvascular permeability (2, 14, 15). By aiding in lymphatic drainage, VEGF-D helps maintain tissue-fluid homeostasis (16, 17).

These biological effects are particularly relevant in the context of SARS-CoV-2-associated disease, especially multisystem inflammatory syndrome in children (MIS-C), which is characterized by systemic inflammation, endothelial injury, microcirculatory dysfunction, capillary leakage, and multisystem involvement (18, 19). Therefore, VEGF-D was selected as a candidate marker reflecting the lymphangiogenic–endothelial component of SARS-CoV-2-associated vascular injury rather than as a nonspecific indicator of overall VEGF-pathway activation.

The relevance of VEGF signaling in SARS-CoV-2-associated disease is further supported by evidence linking hypoxemia, cytokine activation, and progressive lung injury with vascular dysfunction and disruption of endothelial barrier integrity (20). In recent years, VEGF-D has gained attention as a potential biomarker for assessing the clinical course and severity stratification of COVID-19, particularly in adult cohorts (21). This interest is also relevant in pediatrics, where severe acute COVID-19 and MIS-C involve overlapping inflammatory and vascular mechanisms. However, available pediatric evidence has mainly focused on total VEGF in SARS-CoV-2-associated pneumonia and VEGF-A in MIS-C, whereas the specific contribution of VEGF-D remains insufficiently characterized (22, 23).

In particular, changes in VEGF-D levels have been suggested as a way to differentiate between critical and non-critical cases of COVID-19 in adult populations (21). Additionally, VEGF-D has been considered a marker associated with endothelial dysfunction, hypoxia, and pathological angiogenesis, all of which are characteristic of severe COVID-19. In an adult COVID-19 cohort, VEGF-D demonstrated higher diagnostic accuracy than D-dimer and correlated with the extent of organ dysfunction measured by the Sequential Organ Failure Assessment (SOFA) score (21). At the same time, VEGF-A has also been extensively investigated in COVID-19. A recent systematic review and meta-analysis including 11 studies and 1119 patients demonstrated that elevated VEGF-A levels were significantly associated with COVID-19 severity, supporting the prognostic relevance of VEGF-A in COVID-19 (24).

At hospital admission, elevated levels of VEGF in adults with COVID-19 have been linked to more severe disease, including ICU admission, increased risk of acute respiratory distress syndrome (ARDS), acute kidney injury, shock, and death (25, 26). In the context of SARS-CoV-2 infection, VEGF-D is particularly relevant because severe COVID-19 and MIS-C are characterized by hypoxia, systemic inflammation, endothelial dysfunction, and microvascular injury (18, 27–29). These findings are also relevant in pediatrics, as children can also experience severe acute SARS-CoV-2 infection and multisystem inflammatory syndrome in children (MIS-C), which is characterized by involvement of multiple organs and hemodynamic instability that can progress to shock. A study in Ukraine involving adults with COVID-19 showed that the clinical course was associated with specific threshold values of total VEGF, including cutoffs that were linked to an unfavorable course at admission and on day 7 of treatment, as well as a cutoff associated with an increased risk of thrombotic complications (16). Similarly, studies in children have demonstrated that total VEGF levels in SARS-CoV-2-associated pneumonia increase with disease severity and are correlated with higher levels of proinflammatory markers, such as C-reactive protein, procalcitonin, interleukin-1, and interleukin-6 (22). In addition, Gelzo et al. reported significantly higher serum VEGF-A levels in children with MIS-C at hospital admission compared with healthy controls, supporting the involvement of VEGF-related endothelial and angiogenic pathways in MIS-C (23).

Taken together, current evidence supports the involvement of VEGF-related endothelial pathways in COVID-19 and MIS-C, but the pediatric literature remains largely centered on total VEGF or VEGF-A. The specific contribution of VEGF-D, particularly in MIS-C, remains insufficiently characterized. Because VEGF-D links VEGFR-3-mediated lymphangiogenesis with endothelial remodeling and microvascular permeability, its assessment may provide complementary information on the lymphangiogenic–endothelial component of SARS-CoV-2-associated vascular injury. However, VEGF-D should be interpreted within a broader inflammatory–endothelial context rather than as a substitute for other VEGF-family biomarkers. At the same time, focusing on VEGF-D alone may reduce sensitivity compared with total VEGF or combined VEGF-family panels, which could capture a broader spectrum of angiogenic, lymphangiogenic, and endothelial responses.

Therefore, the objective of this study was to assess the diagnostic usefulness of VEGF-D in severe pediatric COVID-19 and MIS-C, determine clinically relevant cutoff values for these conditions, and examine the relationship between VEGF-D and laboratory markers of systemic inflammation (C-reactive protein, procalcitonin, ferritin, IL-1β, IL-6, TNF-α), as well as clinical indicators of disease severity, such as length of hospital stay, need for ICU care, presence of pneumonia, and hypoxemia as measured by peripheral oxygen saturation (SpO_2_).

## 2 Materials and methods

### 2.1 Study design and participants

The study included 200 children with coronavirus disease 2019 (COVID-19) confirmed by polymerase chain reaction (PCR) or a rapid antigen test, 40 children with multisystem inflammatory syndrome in children (MIS-C), and 24 children without evidence of SARS-CoV-2 infection. SARS-CoV-2 infection was established based on PCR testing and/or a positive antigen test.

Written informed consent for participation was obtained from all parents or legal guardians. The study protocol was approved by the institutional Bioethics Committee (Protocol No. 71, October 25, 2022).

### 2.2 Case definitions and clinical classification

MIS-C was diagnosed according to World Health Organization (WHO) criteria, which include: age 0–19 years; fever lasting ≥3 days; and at least two of the following clinical features: (i) rash, bilateral non-purulent conjunctivitis, or signs of mucocutaneous inflammation; (ii) hypotension or shock; (iii) features of cardiac involvement (myocarditis, pericarditis, valvulitis, or coronary abnormalities) and/or elevated troponin or NT-proBNP; (iv) coagulopathy (elevated D-dimer, prolonged prothrombin time, or activated partial thromboplastin time); (v) acute gastrointestinal symptoms (diarrhea, vomiting, abdominal pain). Mandatory criteria also included evidence of systemic inflammation (elevated erythrocyte sedimentation rate, C-reactive protein (CRP), or procalcitonin), exclusion of other plausible causes of inflammation (including bacterial sepsis and staphylococcal or streptococcal toxic shock syndrome), and evidence of SARS-CoV-2 infection (positive PCR, antigen test, or serology) or documented close contact with a COVID-19 case within the preceding 4 weeks (30).

COVID-19 severity was stratified according to WHO criteria. Mild disease was defined as clinical symptoms of COVID-19 without signs of pneumonia or hypoxemia. Moderate disease was defined as pneumonia with typical clinical manifestations (fever, cough, dyspnea, tachypnea, retractions), with peripheral oxygen saturation (SpO_2_) remaining ≥90% while breathing room air. Severe COVID-19 was defined as severe pneumonia with development of respiratory distress syndrome, SpO_2_ <90% on room air, or the presence of danger signs such as altered consciousness, seizures, or inability/refusal to feed or drink (31).

COVID-19 severity classification and MIS-C diagnosis were established after completion of the initial clinical and laboratory evaluation.

Oxygen therapy was required in 27.27% of children with severe COVID-19 and in 7.5% of children with MIS-C. No patient required invasive mechanical ventilation, extracorporeal membrane oxygenation (ECMO), or other advanced life-support modalities. No deaths were recorded, and all patients survived to hospital discharge.

### 2.3 Age and sex characteristics

The age and sex distribution of the study groups is presented in **Table 1**. No statistically significant differences in sex distribution were observed between groups (p>0.05). Age-related analyses were performed using the Pediatric Terminology age-period classification proposed by the National Institute of Child Health and Human Development (32). Participants were categorized into the following age groups: infants (0–12 months), toddlers (1–2 years), early childhood (2–5 years), middle childhood (6–11 years), and early adolescence (12–17 years). The age distribution differed across study groups; children aged <1 year were observed exclusively in the acute COVID-19 group and were not represented among MIS-C patients or controls (**Table 1**).

**Table 1:** Age and sex characteristics of the study groups.

Parameter	COVID-19	MIS-C	Control	χ^2^, p
Sex				
Male	103 (51.50)	24 (60.00)	12 (50.00)	χ^2^ = 1.04, p=0.594
Female	97 (48.50)	16 (40.00)	12 (50.00)	
Age groups				
Infancy	56 (28.00)	−	−	χ^2^ = 36.01, p<0.001*
Toddler	21 (10.50)	5 (12.50)	−	
Early childhood	47 (23.50)	12 (30.00)	8 (33.33)	
Middle childhood	34 (17.00)	17 (42.50)	7 (29.17)	
Early adolescence	42 (21.00)	6 (15.00)	9 (37.50)	

*statistically significant result.

### 2.4 Laboratory measurements

Venous blood samples were collected within the first 24 hours after hospital admission and prior to initiation of pharmacotherapy in order to minimize the potential treatment-related effects on laboratory parameters.

Serum VEGF-D concentrations were measured using an enzyme-linked immunosorbent assay (ELISA) with the Human VEGF-D (Vascular Endothelial Growth Factor D) ELISA Kit (E-EL-H1601), in accordance with the manufacturer’s instructions. The analytical detection range of the VEGF-D ELISA kit was 62.5–4000 pg/mL. Samples with VEGF-D concentrations exceeding the upper calibration limit were diluted and re-assayed according to the manufacturer’s instructions. Final VEGF-D concentrations were calculated using the corresponding dilution factor, and dilution-corrected values were used for statistical analysis.

VEGF-D levels were analyzed according to the predefined clinical groups; therefore, the study assessed diagnostic performance rather than longitudinal prognostic prediction.

Acute-phase markers were determined by ELISA using commercial kits: Human CRP (C-Reactive Protein) ELISA Kit (Cat. No. E-EL-H0043) (Elabscience, USA), Human PCT (Procalcitonin) ELISA Kit (Cat. No. E-EL-H1492) (Elabscience, USA), and Ferritin AccuBind ELISA Kit (Cat. No. 2825-300A) (Monobind Inc., USA). Serum cytokine levels were quantified by ELISA using Elabscience kits according to the manufacturers’ protocols: Human IL-1β (Interleukin 1 Beta) ELISA Kit (Cat. No. E-EL-H0149), Human IL-6 (Interleukin 6) ELISA Kit (Cat. No. E-EL-H6156), and Human TNF-α (Tumor Necrosis Factor Alpha) ELISA Kit (Cat. No. E-EL-H0109).

### 2.5 Statistical analysis

Statistical and graphical analyses were performed using GraphPad Prism 8.4.3, Statistica 13.0 (StatSoft Inc., Tulsa, OK, USA), and JASP 0.19.3. Given the non-normal distribution of quantitative variables, between-group comparisons were conducted using the nonparametric Mann–Whitney U test and Kruskal–Wallis test, as appropriate. Multivariable logistic regression analysis was performed to identify independent markers associated with severe COVID-19 and MIS-C.

Receiver operating characteristic (ROC) analysis was used to evaluate the discriminatory performance of the logistic models by the area under the curve (AUC) with 95% confidence intervals (CI), based on predicted probabilities for: (1) severe COVID-19 versus non-severe disease (mild + moderate) and (2) MIS-C versus COVID-19; AUC significance was assessed relative to 0.5. ROC analysis was also used to determine VEGF-D diagnostic (cutoff) values. Statistical significance was set at p<0.05.

Associations between variables were assessed using Spearman’s rank correlation. Principal component analysis (PCA) was applied to construct an integrated index reflecting shared variation between VEGF-D and systemic inflammatory markers. Due to the skewed distribution of the original data, ln-transformed variables were entered into the PCA: ln(VEGF-D), ln(CRP), ln(ferritin), ln(procalcitonin), ln(IL-1β), ln(IL-6), and ln(TNF-α). Sampling adequacy for PCA was evaluated using the Kaiser–Meyer–Olkin (KMO) measure and Bartlett’s test of sphericity. The number of components retained was determined by parallel analysis. PCA-derived indices were compared between clinical groups using Student’s t test for independent samples (non-severe vs severe COVID-19; COVID-19 vs MIS-C). Effect size was quantified using Hedges’ g; two-sided p values <0.05 were considered statistically significant.

## 3 Results

### 3.1 VEGF-D concentrations in COVID-19, MIS-C, and controls

**Figure 1:**
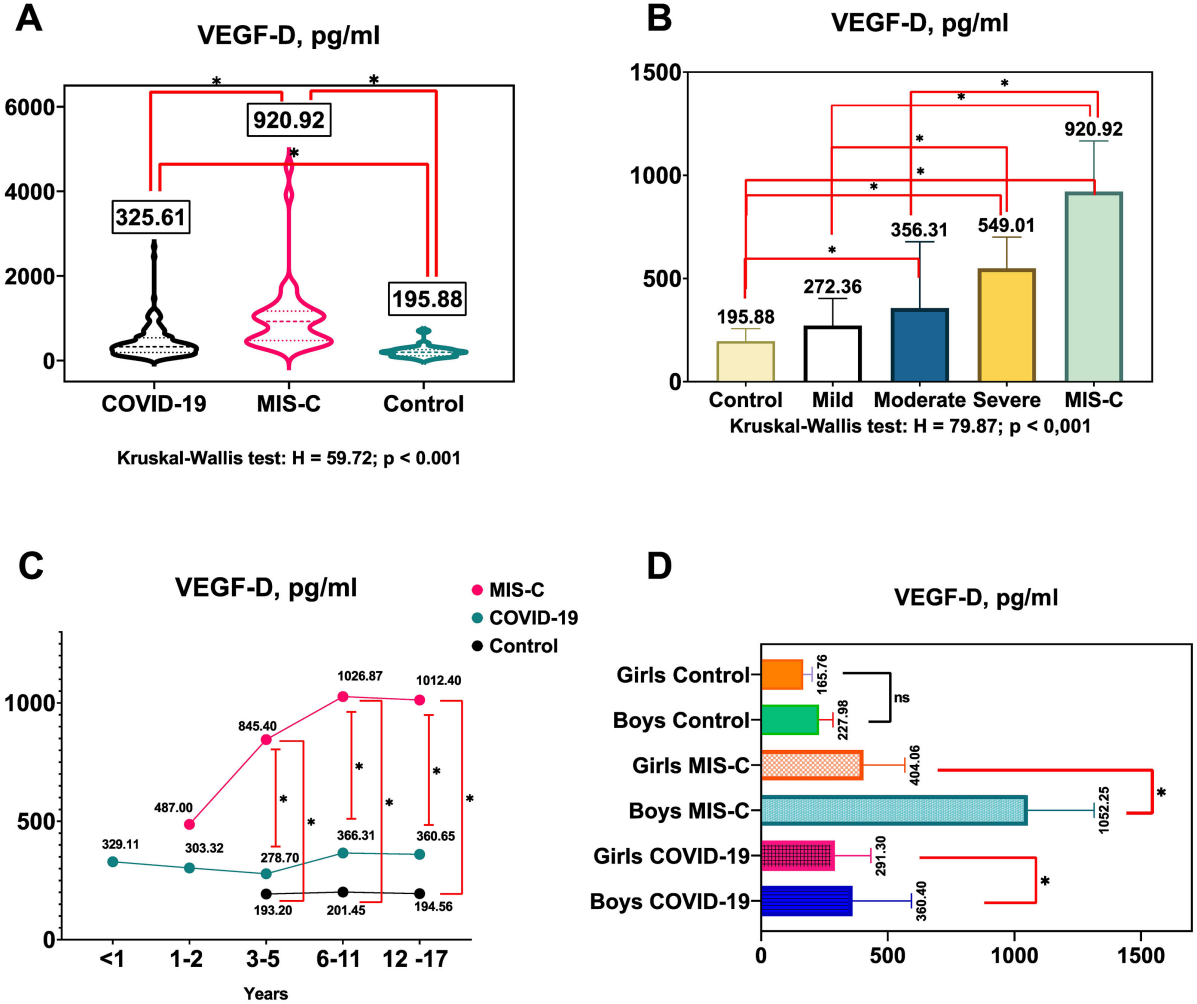
Comparative analysis of VEGF-D levels in children with COVID-19 and MIS-C and in healthy controls, stratified by disease severity, age, and sex. 1. **(A)** COVID-19 vs MIS-C vs controls. **(B)** Controls, COVID-19 (mild/moderate/severe), and MIS-C. **(C)** Age groups. **(D)** Sex subgroups. 2. Data are presented as median [Q1; Q3]. 3. Overall between-group differences were assessed using the Kruskal–Wallis test. 4. VEGF-D, vascular endothelial growth factor D; MIS-C, multisystem inflammatory syndrome in children. 5. *statistically significant result; ns, not significant.

VEGF-D levels in patients with acute SARS-CoV-2 infection (COVID-19) and MIS-C were significantly higher than those observed in the control group (**Figure 1**). Children with MIS-C had approximately threefold higher VEGF-D concentrations than children with COVID-19 (920.92 [473.45; 1157.70] pg/mL vs 325.61 [189.93; 535.85] pg/mL; p<0.05). Notably, VEGF-D levels did not differ significantly between patients with severe COVID-19 and those with MIS-C. In contrast, VEGF-D concentrations in children with mild and moderate COVID-19 were significantly lower than in the MIS-C group (p<0.05). Severe COVID-19 was also associated with significantly higher VEGF-D levels compared with mild disease (**Figure 1**).

### 3.2 Sex- and age-related patterns of VEGF-D

Sex-stratified analyses demonstrated significantly higher VEGF-D levels in boys than in girls in both the COVID-19 and MIS-C groups. No sex-related differences in VEGF-D were observed among uninfected controls (**Figure 1**). Importantly, among girls, VEGF-D levels did not differ significantly between the COVID-19 and MIS-C groups, whereas among boys, VEGF-D concentrations were markedly higher in MIS-C than in COVID-19 (**Figure 1**).

Within-group analyses showed no significant age-related differences in VEGF-D concentrations (COVID-19: H = 1.96, p=0.743; MIS-C: H = 3.11, p=0.375; controls: H = 0.02, p=0.992) (**Figure 1**). However, between-group comparisons within age strata indicated that in children aged 3–5, 6–11, and 12–17 years, VEGF-D levels were significantly higher in the MIS-C group than in both the COVID-19 and control groups (p<0.05). No significant between-group differences were identified in the 1–2-year age stratum (**Figure 1**).

### 3.3 VEGF-D as an independent predictor of severe COVID-19 and MIS-C

Given the observed differences in VEGF-D levels across study groups according to clinical phenotype and disease severity, as well as age- and sex-related patterns, multivariable logistic regression was performed to evaluate the independent association between VEGF-D and the risk of severe COVID-19 and MIS-C in children, adjusting for age and sex. **Table 2** summarizes the regression models for severe COVID-19 and MIS-C.

**Table 2:** Multivariable logistic regression analysis of the association between VEGF-D levels and the risk of severe COVID-19 and MIS-C in children, adjusted for age and sex.

Outcome	Variable	Estimate	Standard error	z	p	Odds ratio (95% CI)
Severe COVID-19	Intercept	-6.74	1.28	5.27	<0.001*	−
	Age	0.54	0.18	2.90	0.004*	1.71 (1.21−2.52)
	Sex	1.18	0.53	2.22	0.027*	3.25 (1.19−9.80)
	VEGF-D	0.002	0.0005	3.46	0.0005*	1.002 (1.001−1.003)
MIS-C	Intercept	-3.59	1.19	3.01	0.003*	−
	Age	0.34	0.20	1.73	0.083	1.41 (0.97−2.12)
	Sex	-0.78	0.61	1.28	0.201	0.46 (0.12−1.43)
	VEGF-D	0.002	0.0005	3.63	0.0003*	1.002 (1.001−1.003)

*statistically significant result .

Severe COVID-19 was significantly associated with sex, age, and VEGF-D level (all p<0.05). Specifically, boys had 3.25-fold higher odds of severe COVID-19 than girls (OR = 3.25; 95% CI 1.19–9.80; p=0.027). Increasing age was also associated with a higher risk of severe disease: each increment to the next older age category increased the odds of severe COVID-19 by 1.71-fold (OR = 1.71; 95%CI 1.21–2.52; p=0.004). Higher VEGF-D levels were likewise associated with increased odds of severe COVID-19 (OR = 1.002 per 1 pg/mL; p<0.001), corresponding to an approximate 20–22% increase in odds per 100 pg/mL increase in VEGF-D (**Table 2**).

VEGF-D remained the only variable independently associated with MIS-C (OR = 1.002 per 1 pg/mL; 95% CI 1.001–1.003; p=0.0003), whereas age and sex were not significant covariates (**Table 2**).

The models demonstrated acceptable discriminatory performance. For severe COVID-19, the area under the ROC curve (AUC) was 0.835 (standard error 0.035; 95% CI 0.767–0.903; p<0.0001). For MIS-C, the AUC was 0.830 (standard error 0.051; 95% CI 0.730–0.930; p<0.0001) (**Table 2**).

### 3.4 Diagnostic performance of VEGF-D: cutoff determination and clinical and laboratory correlates

**Figure 2:**
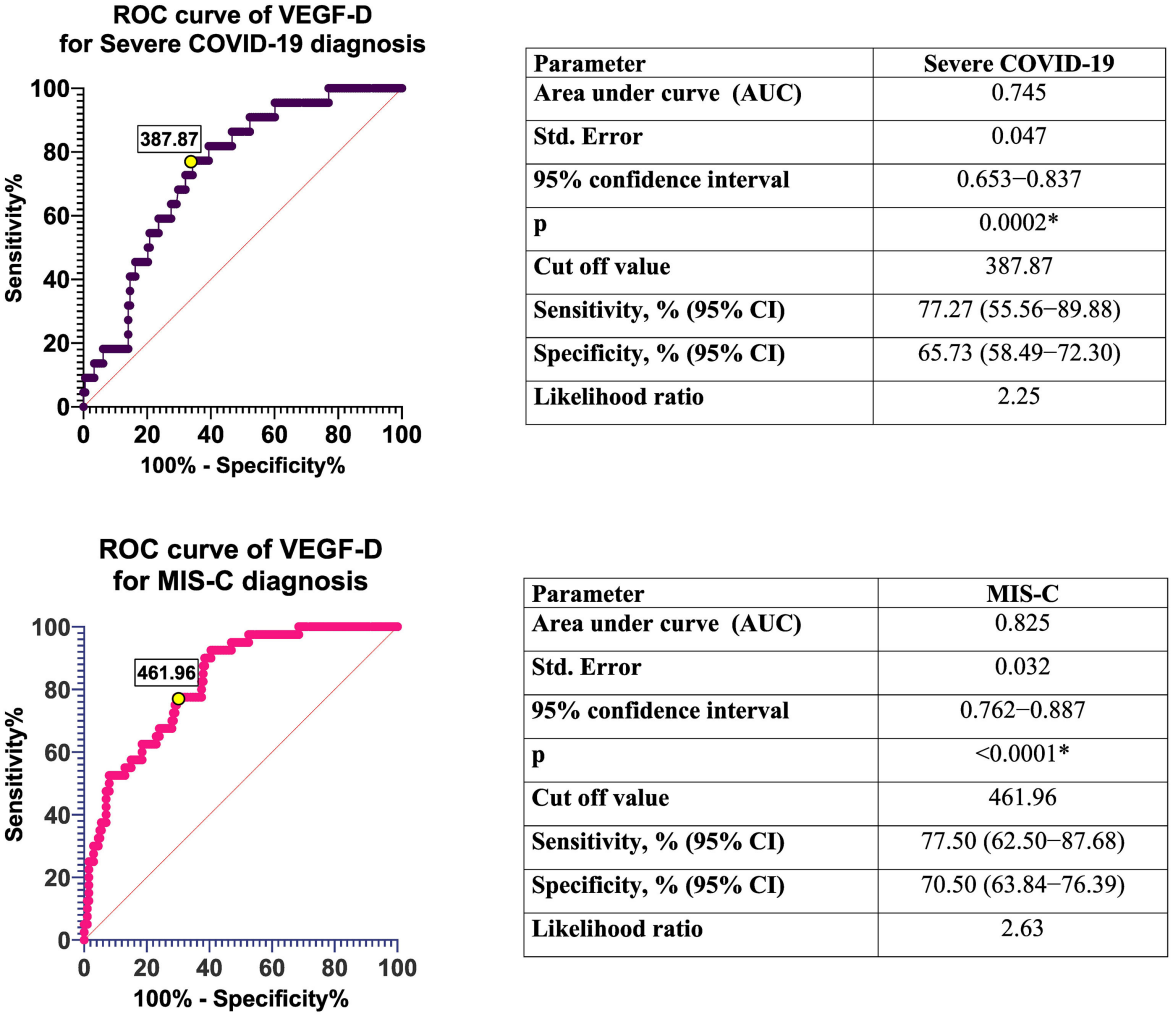
Receiver operating characteristic (ROC) analysis for determining VEGF-D cut-off values for severe COVID-19 in children and for discriminating MIS-C from COVID-19. 1. VEGF-D, vascular endothelial growth factor D; MIS-C, multisystem inflammatory syndrome in children; ROC, receiver operating characteristic; AUC, area under the curve; CI, confidence interval. 2. *statistically significant result.

Receiver operating characteristic (ROC) analysis was performed to determine clinically relevant VEGF-D cut-off values for potential diagnostic application. The optimal VEGF-D threshold for identifying severe COVID-19 was 387.87 pg/mL (AUC = 0.745; sensitivity, 77.27%; specificity, 65.73%). For discriminating MIS-C from acute COVID-19, the optimal cut-off value was 461.96 pg/mL (AUC = 0.825; sensitivity, 77.50%; specificity, 70.50%) (**Figure 2**).

Clinical associations between VEGF-D and severity-related indicators (length of hospital stay, ICU admission, pneumonia, and hypoxemia) were evaluated. Hypoxemia was assessed by peripheral oxygen saturation (SpO_2_).

Length of hospitalization in patients with MIS-C was more than twofold longer than in children with acute SARS-CoV-2 infection. In the COVID-19 group, VEGF-D demonstrated a weak positive correlation with length of stay (r_s_=0.16; p=0.022), whereas no correlation was observed in MIS-C (r_s_=−0.08; p=0.630) (**Table 3**).

**Table 3:** Clinical and laboratory determinants of disease severity and their associations with VEGF-D in children with COVID-19 and MIS-C.

Indicator	COVID-19	MIS-C	р
Length of hospital stay, days, median (IQR)	6.0 (4.0; 9.0)	14.0 (10.0; 16.5)	p<0.001*
Correlation: VEGF-D vs length of hospital stay (Spearman r_s_)	r_s_ = 0.16; p=0.022*	r_s_ = -0.08; p=0.630	
Pneumonia, n (%)	94 (47.00)	19 (47.50)	p=1.000
SpO_2_ on room air, %, median (IQR)	98.0 (96.0; 98.0)	96.0 (95.0; 97.0)	p<0.001*
Correlation: VEGF-D vs SpO_2_ (Spearman r_s_)	r_s_ = -0.21; p=0.002*	r_s_ = -0.17; p=0.268	
ICU admission, n (%)	9 (4.50)	19 (47.50)	p<0.001*
ICU length of stay, days, median (IQR)	5.0 (2.0; 6.0)	5.0 (3.0; 6.0)	p=0.381
Correlation: VEGF-D vs ICU length of stay (Spearman r_s_)	r_s_ = 0.42; p=0.258	r_s_ = 0.10; p=0.684	
C-reactive protein, mg/L	7.20 (4.15; 12.10)	27.25 (11.65; 49.20)	p<0.001*
Procalcitonin, ng/mL	0.21 (0.11; 0.41)	1.05 (0.62; 2.49)	p<0.001*
Ferritin, ng/mL	82.14 (39.86; 134.75)	210.60 (131.83; 348.20)	p<0.001*
Interleukin-1β, pg/mL	4.01 (1.45; 7.22)	10.20 (7.89; 14.31)	p<0.001*
Interleukin-6, pg/mL	79.64 (33.69; 223.00)	295.40 (120.20; 608.88)	p<0.001*
Tumor necrosis factor-α, pg/mL	32.11 (16.60; 92.42)	112.34 (57.75; 466.30)	p<0.001*

^1^Between-group comparisons (COVID-19 vs MIS-C) were performed using the Mann–Whitney U test for continuous variables and Fisher’s exact test for categorical variables.

^2^Correlations were assessed using Spearman’s rank correlation coefficient (r_s_).

^3^*p < 0.05.

Although the frequency of pneumonia did not differ between groups, within the COVID-19 cohort VEGF-D levels were significantly higher in children with pneumonia than in those without pneumonia (422.38 [265.40; 686.20] pg/mL vs 272.36 [156.83; 401.24] pg/mL; p<0.001). In the MIS-C cohort, VEGF-D concentrations did not differ between patients without focal lung involvement (983.08 [467.60; 1070.00] pg/mL) and those with pneumonia (917.74 [487.00; 1176.40] pg/mL; p=0.946). In acute COVID-19, VEGF-D was significantly associated with lower SpO_2_ (greater hypoxemia), whereas this association was not observed in MIS-C (**Table 3**). Although the median SpO_2_ values in the MIS-C group remained within the normal range, individual patients had episodes of oxygen desaturation. Oxygen therapy was required in 7.5% of children with MIS-C.

No significant correlations were identified between ICU length of stay and VEGF-D in either cohort. However, children requiring ICU care had substantially higher VEGF-D levels than those managed outside the ICU (945.02 [483.15; 1443.00] pg/mL vs 341.70 [201.56; 561.34] pg/mL; p<0.001) (**Table 3**). Acute-phase reactants (CRP, procalcitonin, ferritin) and pro-inflammatory cytokines (IL-1β, IL-6, TNF-α) were also significantly higher in MIS-C than in COVID-19 (all p<0.001) (**Table 3**).

**Figure 3:**
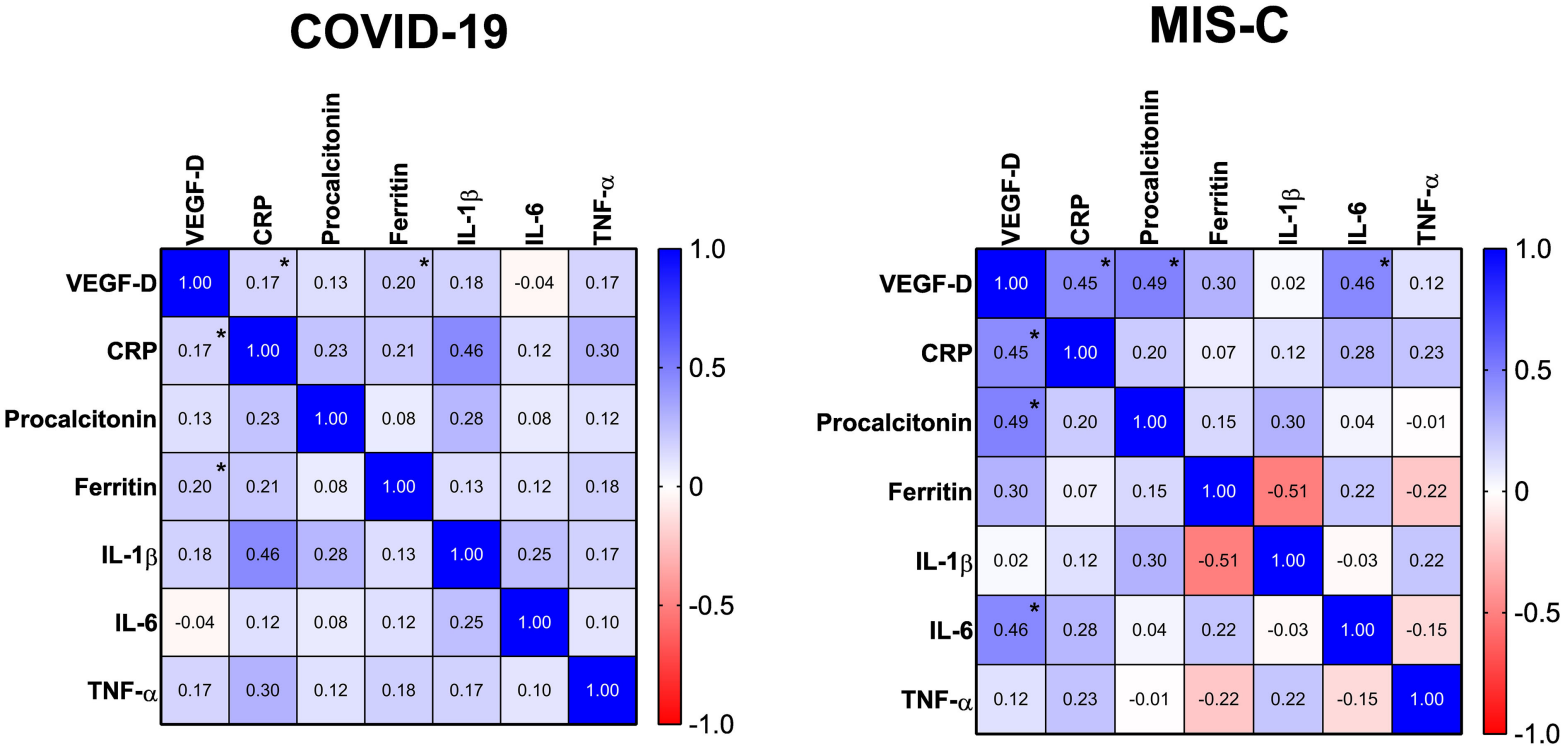
Associations between VEGF-D levels and acute-phase/inflammatory markers in children with COVID-19 and MIS-C. 1. VEGF-D, vascular endothelial growth factor D; MIS-C, multisystem inflammatory syndrome in children; CRP, C-reactive protein; IL-1β, interleukin 1β; IL-6, interleukin 6; TNF-α, tumor necrosis factor α. 2. *statistically significant result.

Spearman correlation analysis between VEGF-D and acute-phase markers demonstrated positive associations between VEGF-D and systemic inflammatory parameters in both study groups (**Figure 3**). In the COVID-19 group, VEGF-D showed significant positive correlations with C-reactive protein (CRP) (r_s_=0.17; p<0.05) and ferritin (r_s_=0.20; p<0.05). In the MIS-C group, VEGF-D was significantly correlated with CRP (r_s_=0.45; p<0.05), procalcitonin (r_s_=0.49; p<0.05), and IL-6 (r_s_=0.46; p<0.05). In contrast, correlations with ferritin, IL-1β, and TNF-α did not reach statistical significance (p>0.05).

**Figure 4:**
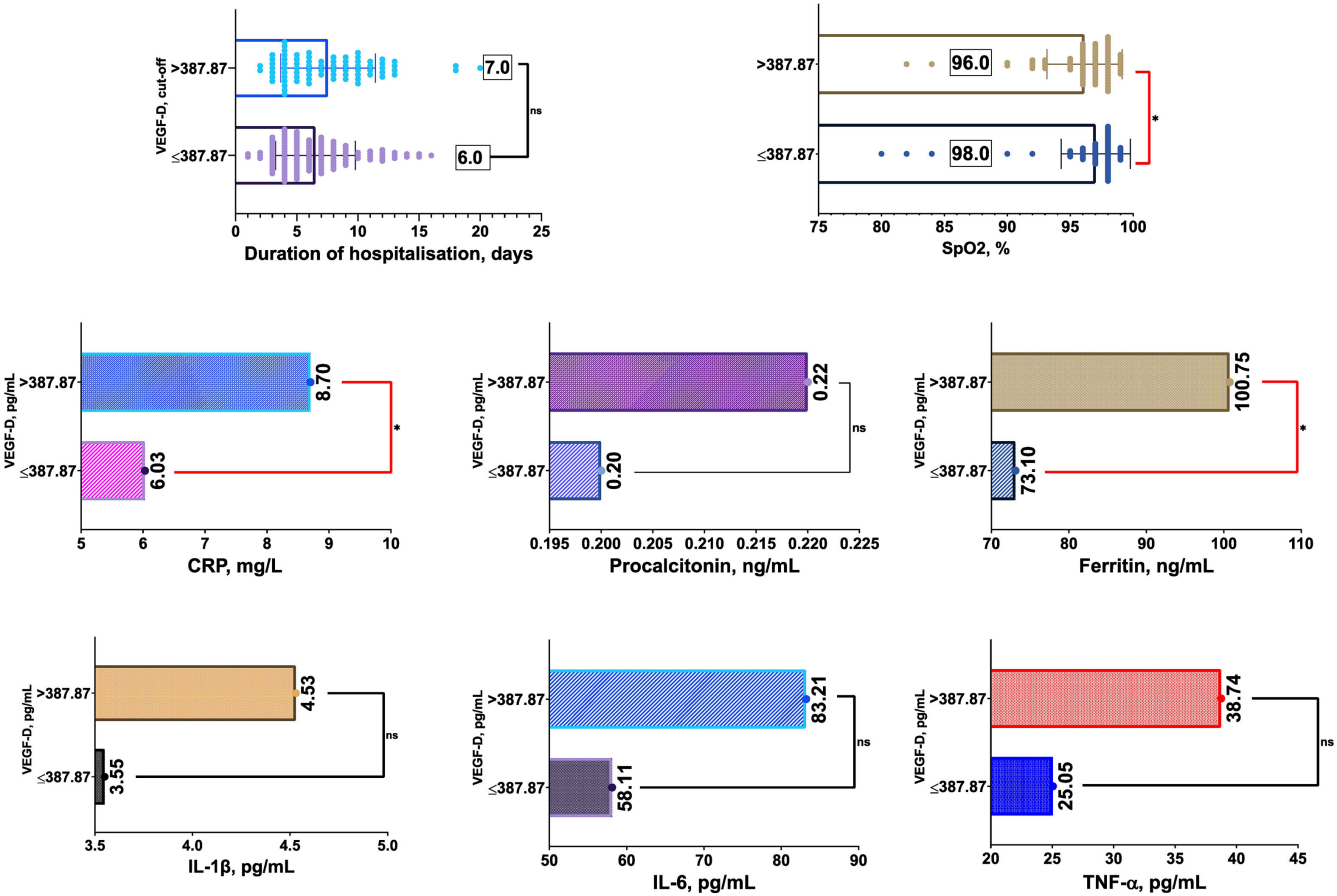
Clinical and laboratory characteristics of pediatric COVID-19 stratified by the VEGF-D cut-off value for severe disease (387.87 pg/mL). 1. VEGF-D, vascular endothelial growth factor D; MIS-C, multisystem inflammatory syndrome in children; CRP, C-reactive protein; IL-1β, interleukin 1β; IL-6, interleukin 6; TNF-α, tumor necrosis factor α. 2. *statistically significant result.

Using the predefined VEGF-D cut-off values for identifying severe COVID-19 (387.87 pg/mL) and for discriminating MIS-C from COVID-19 (461.96 pg/mL), clinical and laboratory parameters were analyzed according to VEGF-D status (**Figure 4**), including length of hospital stay, oxygen saturation (SpO_2_), acute-phase inflammatory markers (CRP, procalcitonin, ferritin) and cytokine profile (IL-1β, IL-6, TNF-α).

In the COVID-19 group, SpO_2_ was significantly lower in children with VEGF-D >387.87 pg/mL compared with those below the cut-off (96.0% vs 98.0%), indicating more pronounced hypoxemia. In contrast, length of hospitalization did not differ between COVID-19 subgroups stratified by VEGF-D. VEGF-D >387.87 pg/mL was also associated with significantly higher CRP (8.70 mg/L vs 6.03 mg/L) and ferritin levels (100.75 ng/mL vs 73.10 ng/mL). By contrast, IL-1β, IL-6, and TNF-α concentrations did not differ between the VEGF-D–stratified COVID-19 subgroups (all p>0.05) (**Figure 4**).

**Figure 5:**
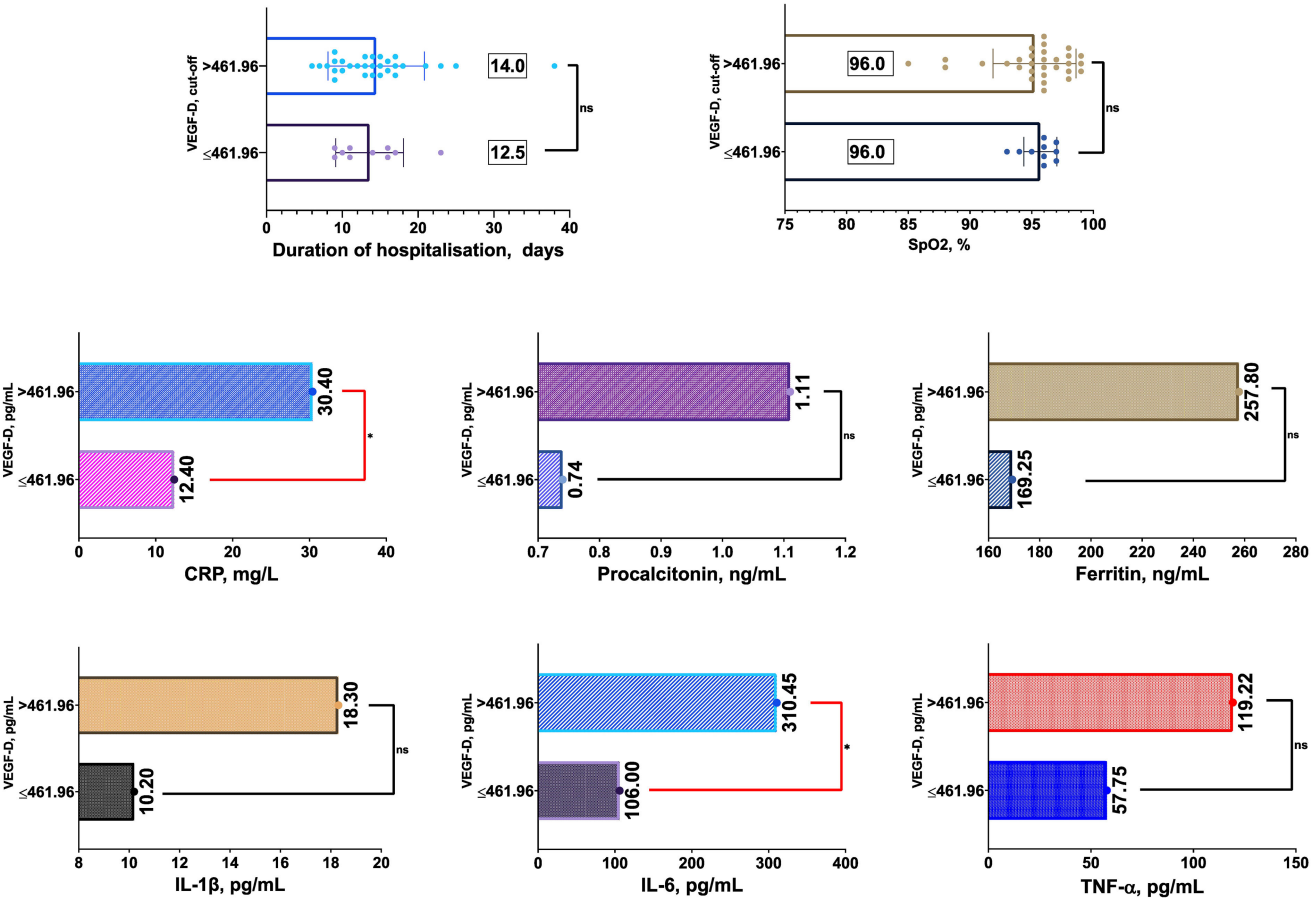
Clinical and laboratory characteristics of the MIS-C cohort stratified by the VEGF-D cut-off value (461.96 pg/mL). 1. VEGF-D, vascular endothelial growth factor D; MIS-C, multisystem inflammatory syndrome in children; CRP, C-reactive protein; IL-1β, interleukin 1β; IL-6, interleukin 6; TNF-α, tumor necrosis factor α. 2. *statistically significant result.

In the MIS-C group, no significant differences in length of hospitalization or SpO_2_ were observed between patients with VEGF-D >461.96 pg/mL and those with VEGF-D ≤461.96 pg/mL (p>0.05). By contrast, CRP and IL-6 levels were significantly higher among MIS-C patients with VEGF-D exceeding the diagnostic cut-off for MIS-C (461.96 pg/mL). Procalcitonin, ferritin, IL-1β, and TNF-α levels were not associated with VEGF-D cut-off status in MIS-C (all p>0.05) (**Figure 5**).

### 3.5 VEGF-D within an integrated inflammatory–endothelial signature: principal component–derived indices

To determine whether VEGF-D contributes to a shared, integrated profile together with markers of systemic inflammation (acute-phase reactants and cytokines), and to derive a composite index associated with disease severity and phenotype of SARS-CoV-2–related conditions in children, principal component analysis (PCA) was performed in two samples: (1) the COVID-19 cohort and (2) the combined COVID-19/MIS-C cohort. The analysis included natural log-transformed biomarkers: ln(VEGF-D), ln(CRP), ln(ferritin), ln(procalcitonin), ln(IL-1β), ln(IL-6), and ln(TNF-α).

**Figure 6:**
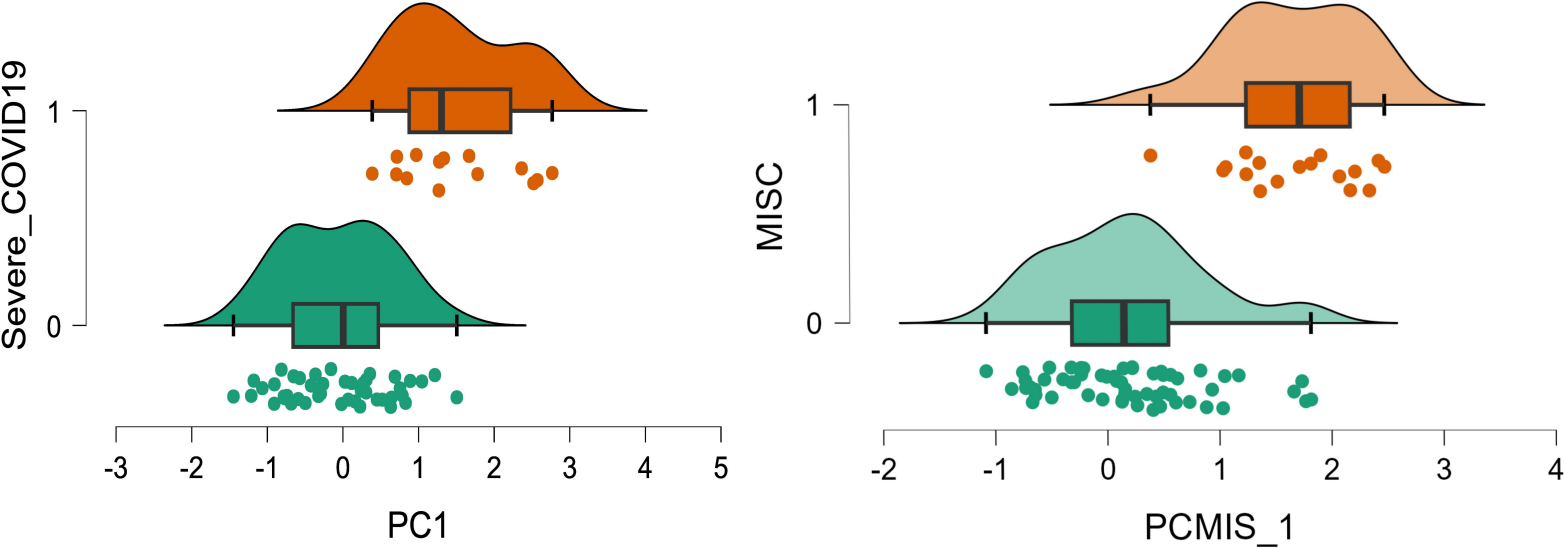
Principal component analysis-derived inflammatory-endothelial profile indices and their associations with COVID-19 severity and MIS-C in children. 1. PC1/PCMIS_1 represents the first principal component derived from ln(VEGF-D), ln(CRP), ln(ferritin), ln(procalcitonin), ln(IL-1β), ln(IL-6), and ln(TNF-α). 2. Dots indicate individual values; boxplots show the median (IQR); density curves depict the distribution. 3. Groups: Severe_COVID19 (0/1) and MIS-C (0/1).

Sampling adequacy for PCA was acceptable (KMO = 0.655), and Bartlett’s test of sphericity indicated sufficient inter-variable correlations (χ²=45.48; p=0.001). Parallel analysis supported retention of a single component. The first principal component (PC1) explained 31.5% of the total variance (eigenvalue=2.20) and showed the highest loadings for ln(CRP) (0.762), ln(IL-1β) (0.626), and ln(VEGF-D) (0.590) (**Table 4**). PC1 was therefore interpreted as an integrated inflammatory–endothelial severity index. PC1 scores were significantly higher in children with severe COVID-19 (1.51 ± 0.79) than in those with non-severe disease (−0.06 ± 0.72) (t=−6.98; p<0.001), with a very large effect size (Hedges’ g=−2.11), indicating marked separation of severity groups based on the integrated profile that includes VEGF-D (**Figure 6**).

**Table 4:** Principal component analysis loading matrix for severity-related biomarkers in pediatric COVID-19 (PC1) and in MIS-C (PCMIS_1).

COVID-19			MIS-C		
Parameter	PC1	Uniqueness	Parameter	PCMIS_1	Uniqueness
ln_CRP	0.762	0.419	ln_CRP	0.858	0.264
ln_IL-1β	0.626	0.608	ln_Procalcitonin	0.762	0.419
ln_VEGF-D	0.590	0.652	ln_IL-6	0.734	0.462
ln_TNF-α	0.581	0.662	ln_TNF-α	0.733	0.462
ln_Ferritin	0.512	0.738	ln_VEGF-D	0.676	0.543
ln_IL-6	0.396	0.844	ln_IL-1β	0.675	0.544
ln_Procalcitonin	0.356	0.874	ln_Ferritin	0.590	0.651

In the combined cohort, the PCA structure was more coherent, with high sampling adequacy (KMO = 0.850) and statistically significant correlations among variables (χ²=207.23; p<0.001). Parallel analysis again supported a single-component solution, with substantially greater explained variance: PC1 accounted for 52.2% of the variance (eigenvalue=3.65), consistent with a more consolidated inflammatory–endothelial pattern. The strongest loadings were observed for ln(CRP) (0.858), ln(procalcitonin) (0.762), ln(IL-6) (0.734), and ln(TNF-α) (0.733). VEGF-D retained a substantial contribution to this integrated profile (ln[VEGF-D]=0.676), comparable to ln(IL-1β) (0.675) (**Table 4**). PCA index values (PCMIS_1; PC1) differed significantly between the COVID-19 and MIS-C groups (t=−8.12; p<0.001; Hedges’ g=−2.22). The mean difference (COVID–MIS-C) was −1.49 ± 0.18, indicating higher PCMIS_1 scores in MIS-C (**Figure 6**).

Across both samples, PCA consistently identified a single dominant component integrating the variability of acute-phase markers and pro-inflammatory cytokines with a meaningful contribution from VEGF-D. These findings support the assessment of VEGF-D alongside conventional inflammatory markers when evaluating severe pediatric COVID-19 and MIS-C, as part of an integrated inflammatory–endothelial profile.

## 4 Discussion

The present study highlights the clinical significance of VEGF-D as a biomarker for stratifying disease severity in pediatric SARS-CoV-2 infection and its potential role in identifying MIS-C.

To place these findings in a broader biological context, the VEGF family has pleiotropic effects on multiple cell types, including endothelial cells, fibroblasts, stem cells, placental cells, neurons, cardiomyocytes, vascular smooth muscle cells, and pericytes (33, 34). Transcript profiling indicates that VEGF is most strongly expressed in lung tissue compared with other organs in which it is detectable, underscoring the relevance of VEGF signaling in respiratory tissue (34).

Mechanistically, VEGF-D binds to VEGFR-2 and VEGFR-3 on endothelial cells, with signaling through VEGFR-3 primarily affecting lymphatic vessels and signaling through VEGFR-2 primarily affecting blood capillaries (1, 14, 16). Additionally, VEGF-D, along with other members of the VEGF family (VEGF-A, VEGF-B, VEGF-C, VEGF-D, and placenta growth factor), plays a role in regulating lung development during embryogenesis, although its specific role has been less extensively studied compared to VEGF-A (2). Both increased and decreased VEGF pathway activity have been linked to disease: excessive signaling is associated with pathological angiogenesis and tumor progression, whereas reduced signaling has been implicated in impaired wound healing and ischemic phenotypes (3). Furthermore, VEGF signaling has been shown to have anti-apoptotic effects (34, 35).

In SARS-CoV-2 infection, endothelial injury disrupts cellular growth and angiogenic processes, leading to dysregulated secretion of multiple growth factors, such as EGF, FGF, PDGF, VEGF, and TGF (26, 35). Hemodynamics also plays a role in the course of SARS-CoV-2 disease: neovascularization and changes in blood flow can either contain the infection or facilitate its spread, potentially altering the dynamics of the infection (36, 37). Within this context, VEGF-driven sprouting angiogenesis is a key mechanism for directing vascular growth in both healthy and injured tissues (36, 38).

During a viral infection, the body’s inflammatory response often leads to a decrease in oxygen levels in the affected area. This decrease in oxygen can be caused by various factors such as tissue swelling, blood clots, and constriction of blood vessels (27, 39, 40). In response to these conditions, the transcription factor HIF-1α becomes activated and moves into the nucleus, where it increases the production of VEGF-A, VEGF-C, and VEGF-D genes (2, 27, 41–43). This increase in VEGF-D can be seen as a beneficial response, as it helps to improve the function of the microcirculatory system by promoting changes in the blood vessels and enhancing lymphatic drainage.

In addition to its angiogenic effects, VEGF signaling also increases vascular permeability through VEGFR-2 by altering endothelial fenestrations and inter-endothelial junctions (15) (44). In infected lung tissue, elevated levels of VEGF may trigger plasma extravasation, resulting in pulmonary edema and creating conditions that promote bacterial and fungal co-infection (41, 45). The extravasated plasma, rich in cytokines, can further exacerbate inflammation by attracting macrophages and neutrophils to the site of injury. Additionally, the coagulation of extravasated plasma can form a gel-like matrix that impairs gas exchange between the alveolar space and surrounding tissues. These plasma clots can also serve as a scaffold for the invasion of newly formed vessels, fibrotic structures, and other cellular elements, which can lead to acute lung injury and interstitial lung disease (41, 46). Other studies have also linked VEGF-related pathways to acute lung injury/ARDS (47). This pathogenic mechanism may explain the elevated levels of VEGF-D observed in children with pneumonia during acute COVID-19, as well as the correlation between higher VEGF-D concentrations and lower oxygen saturation in the pediatric COVID-19 cohort.

In adult COVID-19 cohorts, VEGF-D has been identified as a severity-associated biomarker and was shown to help differentiate critical from non-critical disease (21). This is consistent with our findings, which demonstrated higher VEGF-D levels in children with severe COVID-19 and MIS-C. However, while there have been reported differences in VEGF levels between severe and non-severe cases of COVID-19, several studies have not found VEGF to be a reliable marker of disease severity in adults (48). Other investigations have shown increased VEGF levels in moderate COVID-19 cases, with subsequent decreases in critically ill ICU patients (35). Some authors have even suggested that VEGF may have prognostic value for mortality (49). It is important to note that these studies primarily involved adult cohorts and measured total VEGF levels, rather than specifically looking at VEGF-D. In our study, we found that VEGF-D was independently associated with severe acute SARS-CoV-2 infection and MIS-C in children, even after adjusting for age and sex.

Taken together, these findings suggest that VEGF-related biomarkers in COVID-19 should be interpreted according to the specific VEGF-family member measured and the clinical context of the cohort. This is particularly relevant when comparing our results with those of Dudnyk and Mykytiuk, who evaluated total VEGF in children with SARS-CoV-2-associated pneumonia, whereas the present study measured VEGF-D specifically in children with acute COVID-19 and MIS-C (22). Therefore, these values are not directly interchangeable: total VEGF reflects a broader angiogenic response, while VEGF-D may more specifically reflect lymphangiogenic–endothelial remodeling.

Differences in circulating SARS-CoV-2 variants may also have contributed to discrepancies between studies. Our study was conducted between 2021 and 2023, a period during which different SARS-CoV-2 variants circulated globally and in Ukraine. SARS-CoV-2 variants differ in transmissibility, immune escape potential, and clinical impact. In Ukraine, genomic surveillance data showed that the third epidemic wave in 2021 was dominated by the Delta variant of concern, whereas surveillance data from May 2022 to March 2024 demonstrated the dynamics and predominance of Omicron-lineage variants (50, 51). However, SARS-CoV-2 variant identification was not performed at the individual-patient level in the present cohort. Therefore, although differences in circulating variants may have influenced inflammatory responses and VEGF-D concentrations, this factor could not be directly assessed in our study.

Altered expression of angiotensin-converting enzyme 2 (ACE2) during SARS-CoV-2 infection may have implications for VEGF signaling. ACE2 has been reported to suppress the VEGF-A/VEGFR2/ERK pathway and to antagonize VEGF-A-mediated vascular permeability; therefore, altered ACE2 signaling may weaken this counter-regulatory effect and contribute to endothelial injury and increased vascular permeability, potentially promoting a more severe COVID-19 course (52, 53). Consistent with this broader ACE2–VEGF interaction, our cohort showed higher VEGF-D levels in both acute SARS-CoV-2 infection and MIS-C than in uninfected controls, with concentrations increasing with clinical severity and reaching the highest values in MIS-C.

When interpreted against age- and sex-specific reference ranges reported by Arélin et al. for healthy children and adolescents aged 0.25–18 years (54), the median VEGF-D concentration in our uninfected participants corresponded approximately to the 10th percentile in the 3–5-year group, the 50th percentile in the 6–11-year group, and the 10th–50th percentile range among adolescents. In contrast, median VEGF-D values in children with COVID-19 exceeded the 50th percentile, whereas those in MIS-C were above the 97.5th percentile. This marked shift from expected age-related levels supports the presence of a pathological inflammatory-endothelial phenotype associated with severe SARS-CoV-2–related disease and multisystem involvement.

No significant differences in VEGF-D were observed between sexes in the healthy control group, which is consistent with typical patterns for both prepubertal and pubertal ages. Similar findings were reported in a cohort of healthy European children living in Germany, where sex differences in VEGF-D were only evident in the postpubertal period (Tanner stage V), with significantly higher concentrations in girls (54). These patterns, however, pertain to healthy individuals without SARS-CoV-2 infection. In our study, VEGF-D levels were significantly higher in boys with COVID-19 and MIS-C, and the magnitude of sex-related differences tended to increase. This finding suggests that SARS-CoV-2 infection and the associated hyperinflammatory state may modify age- and sex-dependent regulation of VEGF-D, resulting in a more pronounced sex-specific response that may be linked to a more severe clinical course in boys.

The marked elevation of VEGF-D in MIS-C may be related to cytokine storm biology and activation of pro-inflammatory cytokine pathways. Specifically, IL-1β, via NF-κB–dependent signaling, has been reported to induce VEGF-D expression and promote lymphangiogenesis (55) Conversely, reduced VEGF-D expression in response to IL-1β stimulation has also been described, including in cardiac microvascular endothelial cells, underscoring the tissue-specific nature of VEGF-D–IL-1β interactions (56). Given the frequent cardiac involvement in MIS-C, this apparent discrepancy may have clinical relevance and warrants further investigation. Evidence from oncologic models further suggests that TNF-α can increase VEGF-D promoter activity through the ERK1/2–AP-1 axis (57), whereas IL-6 may upregulate VEGF-D expression via STAT3 signaling (58).

VEGF signaling has been discussed within a closely interconnected inflammatory framework alongside interleukin cascades, particularly IL-1, IL-6, and IL-17, in MIS-C (59). Moreover, VEGF has been classified among pro-inflammatory mediators together with interleukins (e.g., IL-1β, IL-13, IL-4), which may contribute to myocardial remodeling by promoting differentiation of cardiac fibroblasts into myofibroblasts, thereby underscoring the functional coupling between VEGF pathways and interleukin-driven responses in MIS-C (59). In line with this concept, Isaza-Correa et al. reported elevated levels of both total VEGF and pro-inflammatory cytokines, including IL-1β, IL-6, and TNF-α, in children with MIS-C (60). Our findings are consistent with these observations while specifically highlighting VEGF-D as a component of the inflammatory–endothelial mechanisms implicated in MIS-C.

Whether elevated VEGF-D represents a consequence of MIS-C or contributes to its pathogenesis cannot be determined from the present study because of its cross-sectional design and single-time-point measurement. Increased VEGF-D may reflect a downstream response to MIS-C-related systemic inflammation, cytokine activation, hypoxia, and endothelial injury, indicating activation of lymphangiogenic and endothelial remodeling pathways. At the same time, VEGF-D may also contribute to MIS-C pathophysiology by promoting lymphangiogenesis, microvascular permeability, tissue edema, and immune-cell trafficking. Therefore, VEGF-D should be interpreted as a marker of inflammatory–endothelial activation and a potential contributor to vascular involvement in MIS-C, although longitudinal and mechanistic studies are required to clarify causality.

Several studies of the convalescent phase after acute COVID-19 have reported persistently elevated VEGF-D levels even 3–4 months after infection (5, 61). Such persistence may reflect prolonged endothelial activation and/or dysregulation of angiogenic and lymphangiogenic pathways following the acute illness. MIS-C, however, is a delayed post-infectious syndrome that typically manifests weeks after SARS-CoV-2 infection; therefore, elevated VEGF-D in MIS-C may be interpreted within the framework of a post-infectious immunoinflammatory response and suggests that VEGF-associated perturbations can extend beyond the acute phase. At present, dedicated data describing VEGF-D dynamics in pediatric populations remain limited; in contrast, significantly higher VEGF-A levels have been reported in MIS-C compared with hemophagocytic lymphohistiocytosis (62).

Circulating VEGF levels should also be interpreted cautiously, as plasma concentrations may not fully reflect tissue-level VEGF activity (63). Moreover, most VEGF isoforms are heparin-binding and can be sequestered within tissues, which may influence measured circulating VEGF-D concentrations (63).

## 5 Conclusions

The present study demonstrates characteristic patterns of VEGF-D alterations in children with acute SARS-CoV-2 infection and MIS-C. VEGF-D concentrations increased with disease severity and were highest in children with MIS-C, supporting an association between VEGF-D, systemic inflammation, and endothelial injury.

An important outcome of this study is the determination of VEGF-D cut-off values associated with severe COVID-19 and MIS-C, highlighting the potential utility of this biomarker for disease severity stratification and differentiation of SARS-CoV-2-associated clinical phenotypes. The results also reveal an integrated inflammatory–endothelial pattern underlying severe SARS-CoV-2-associated conditions, in which VEGF-D reflects the interplay between systemic inflammation, endothelial dysfunction, and microcirculatory impairment.

From a practical standpoint, the identified VEGF-D profiles may support the rationale for expanding diagnostic and severity-stratification algorithms for the management of pediatric COVID-19 and MIS-C by incorporating VEGF-D together with conventional inflammatory markers. Additionally, the potentially phenotype-specific nature of VEGF-D responses may be useful for guiding closer longitudinal follow-up of children recovering from different SARS-CoV-2-associated clinical phenotypes, supporting timely recognition of possible post-acute complications and facilitating individualized post-acute management.

## Data Availability

The raw data supporting the conclusions of this article will be made available by the authors, without undue reservation.
